# A catalog of annotated high-confidence SNPs from exome capture and sequencing reveals highly polymorphic genes in Norway spruce (*Picea abies*)

**DOI:** 10.1186/s12864-018-5247-z

**Published:** 2018-12-17

**Authors:** Aïda Azaiez, Nathalie Pavy, Sébastien Gérardi, Jérôme Laroche, Brian Boyle, France Gagnon, Marie-Josée Mottet, Jean Beaulieu, Jean Bousquet

**Affiliations:** 1 Canada Research Chair in Forest Genomics, Forest Research Centre, Université Laval, Québec, Québec G1V 0A6 Canada; 2 Institute of Integrative Biology and Systems, Université Laval, Québec, Québec G1V 0A6 Canada; 3 Direction de la recherche forestière, Ministère des Forêts, de la Faune et des Parcs du Québec, 2700 Einstein, Québec, Québec G1P 3W8 Canada

**Keywords:** Conifer, Exome sequencing, Annotated gene SNPs, Illumina Infinium iSelect array, Illumina MiSeq, *Picea abies*, Single nucleotide polymorphism, SNP abundance

## Abstract

**Background:**

Norway spruce [*Picea abies* (L.) Karst.] is ecologically and economically one of the most important conifer worldwide. Our main goal was to develop a large catalog of annotated high confidence gene SNPs that should sustain the development of genomic tools for the conservation of natural and domesticated genetic diversity resources, and hasten tree breeding efforts in this species.

**Results:**

Targeted sequencing was achieved by capturing *P. abies* exome with probes previously designed from the sequenced transcriptome of white spruce (*Picea glauca* (Moench) Voss)*.* Capture efficiency was high (74.5%) given a high level of exome conservation between the two species. Using stringent criteria, we delimited a set of 61,771 high-confidence SNPs across 13,543 genes. To validate SNPs, a high-throughput genotyping array was developed for a subset of 5571 predicted SNPs representing as many different gene loci, and was used to genotype over 1000 trees. The estimated true positive rate of the resource was 84.2%, which was comparable with the genotyping success rate obtained for *P. abies* control SNPs recycled from previous genotyping efforts. We also analyzed SNP abundance across various gene functional categories. Several GO terms and gene families involved in stress response were found over-represented in highly polymorphic genes.

**Conclusion:**

The annotated high-confidence SNP catalog developed herein represents a valuable genomic resource, being representative of over 13 K genes distributed across the *P. abies* genome. This resource should serve a variety of population genomics and breeding applications in Norway spruce.

**Electronic supplementary material:**

The online version of this article (10.1186/s12864-018-5247-z) contains supplementary material, which is available to authorized users.

## Background

Giant leaps have been made recently regarding the sequencing of spruce genomes, resulting in the release of draft genome sequence assemblies for *Picea abies* (Norway spruce) and *Picea glauca* (white spruce) [1–3]. However, owing to the huge size (∼ 20 Gb) and highly repetitive content of spruce genomes, these sequences remain largely fragmented and not suited to develop reliable population genomic tools [4]. Hence, strategies aiming to reduce genome complexity have been deployed in order to sustain the development of such tools in spruces. During the last decade, most resequencing efforts focused on the gene space, using approaches such as cDNA and EST sequencing, RNA-Seq, or exome sequencing to develop genomic resources [4].

The recent advent of high-throughput technologies for the detection and genotyping of single nucleotide polymorphisms (SNPs) has led to a revolution in their use as reliable molecular markers in spruce population genomics. Because of their abundance in spruce exomes, and ongoing reduction in sequencing and genotyping costs, gene SNPs have been used in a vast array of spruce genomic applications, including gene and QTL mapping (e.g. [5–9]), genomic selection (e.g. [10–13]), association mapping and ecological genomic studies (e.g. [6, 14–25]), the management of genetic diversity, and for traceability applications [15, 26–29]. One central feature of spruce gene SNPs is that they are informed markers, given the availability of high-confidence annotated spruce gene catalogs (e.g. [30]), of dense genetic maps including thousands of genes [7, 9], and of large annotated gene expression databases [31, 32]. Moreover, spruce genomes harbor highly syntenic and collinear macrostructures [33–35], thus allowing the transfer of structural information among congeners.

SNP discovery through resequencing and bioinformatic screening has been shown to be efficient to identify large sets of reliable SNPs in transcribed genes [36, 37]. In conifers, these SNPs were usually validated by genotyping subsets of predicted SNPs and assessing their true positive rate with high-density genotyping arrays [26, 38–41]. In spruces, the first extensive gene SNP catalog was developed for *P. glauca* from cDNA sequencing and expressed sequence tags (ESTs) [42]. It first included ~ 12 K high-confidence nonsingleton SNPs encompassing ~ 6.5 K genes [42], which was further extended to ~ 212 K high-confidence nonsingleton SNPs in ~ 13.5 K expressed genes with a true positive rate of 92% [27]. Exome sequencing is another efficient approach to identify gene SNPs in non-model species with large genomes such as spruces [3, 43]. This approach was successfully used in black spruce (*Picea mariana*) to generate a catalog of ~ 97 K high-confidence SNPs encompassing ~ 15 K genes with true positive rate of 96% [41]. In Norway spruce, two SNP resources have been published to date, but their annotation was rather limited and their true positive rate has not been estimated yet [44, 45].

Along with black spruce and white spruce, Norway spruce is ecologically and economically one of the most important conifers worldwide. It is therefore the subject of important tree breeding efforts in various jurisdictions in Europe [46]. While Norway spruce is originally native from Europe, it was introduced in eastern Canada and northeastern United-States early on in the twentieth Century for the production of lumber, pulp and paper [47]. In the province of Québec, Norway spruce is currently the most productive spruce species [48] and more than 200 million Norway spruce seedlings have been planted since 1968 [49]. Due to its high wood quality, the current demand for Norway spruce seedlings in Eastern Canada is substantial, with nearly 10 millions reforested seedlings per year in Québec, New Brunswick and Nova Scotia [49, G. Adams, J.D. Irving Ltd., personal communication). Accordingly, conventional breeding programs have been set up for Norway spruce in Canada (e.g. [49]). In addition, genomic approaches applied to *P. abies* have also been successfully deployed in Europe in order to gain insight into genomic architecture and evolutionary genetics (e.g. [7, 18]). These genomic approaches mainly relied on markers originally developed in *P. glauca*, as *P. glauca and P. abies* gene SNPs were shown to be partly shared by incomplete lineage sorting [50]. Indeed, a survey of ~ 15 K gene SNPs showed that at least 12% of *P. glauca* SNPs were also found in *P. abies* [27]; hence, hundreds of *P. glauca* SNPs were used to help build early on high throughput genotyping arrays for *P. abies* [7, 18]. However, larger arrays of markers need to be interrogated repeatedly in an efficient and uniform way to apply large-scale genomic approaches such as genome-wide association studies (GWAS) or genomic selection (GS). Therefore, developing large annotated and reliable SNP resources specific to Norway spruce appears necessary. Such resource would also be useful to validate data that may be obtained in the future by genotyping-by-sequencing (GbS) approaches, and further increase the number of markers suited for diverse population genomic applications.

Our primary goals were to generate a catalog of annotated high-confidence SNPs covering much of the exome of *P. abies*, and to evaluate the true positive rate for a subset of predicted SNPs using a genotyping array. Given that success rate is usually high when applying exome capture probes to congeneric species [41, 43, 51, 52], we relied on a large set of probes that were successfully transferred from *P. glauca* to *P. mariana* in a previous study [41]. We also used the SNP resource developed herein to survey nucleotide polymorphism through a large part of the *P. abies* exome and identify gene ontologies (GO) and gene families with highest SNP abundance as a proxy for genetic diversity of potential adaptive significance for future studies.

## Results

### Exome capture and sequencing, de novo assembly, and pairwise sequence comparisons

**Fig. 1:**
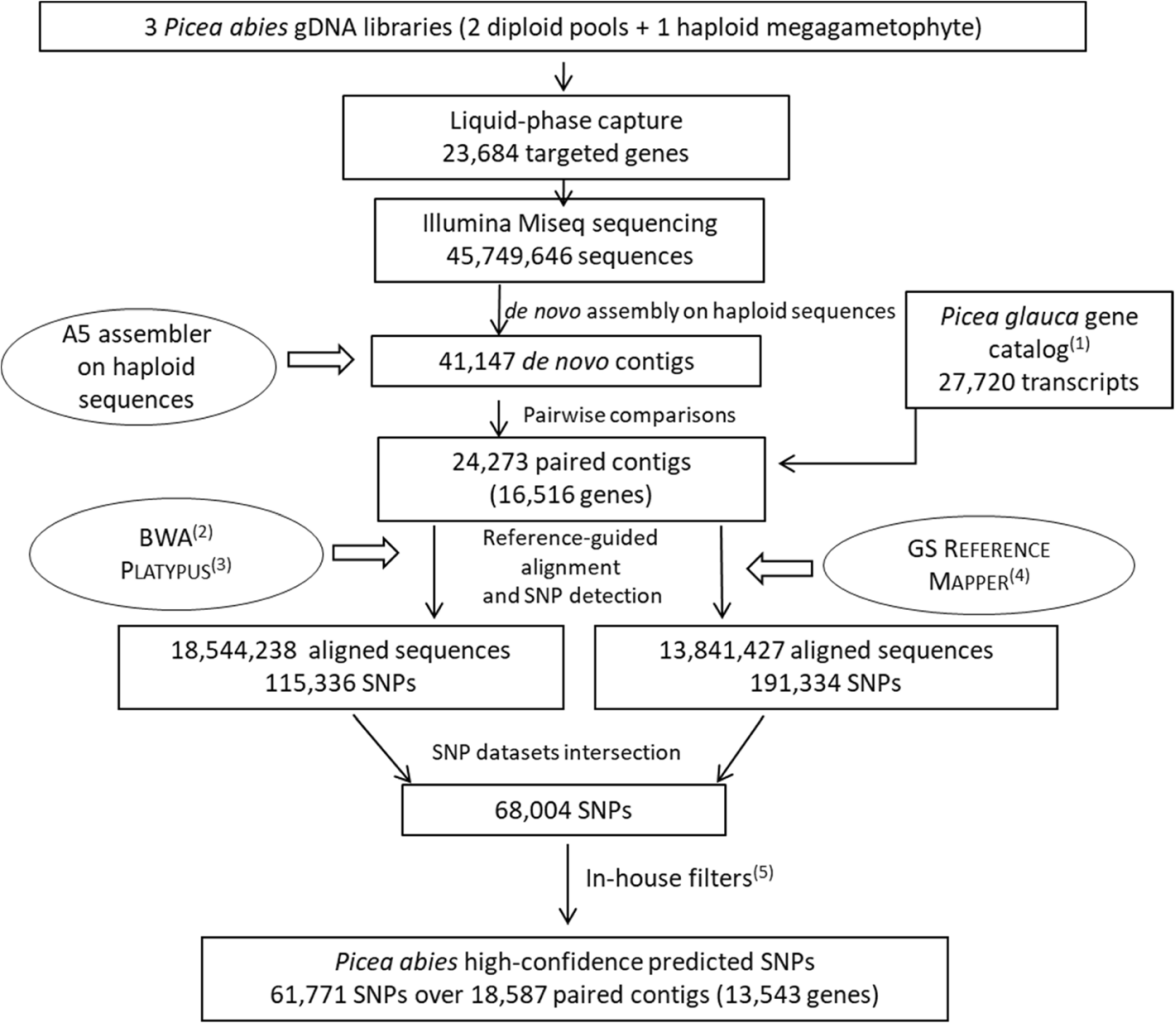
Pipeline for exome capture and sequencing, sequence assembly and SNP discovery. ^1^ [30]. ^2^ [93]. ^3^ [94]. ^4^454 Life Science, Branford, CT.^5 ^See Methods for details

After the liquid-phase capture, Illumina MiSeq sequencing generated two ~ 300-bp paired-end sequences per captured insert, ending with 45,749,646 sequences (Fig. 1). The assembly process resulted in 41,147 de novo contigs longer than 500 bp (average length of 1036 bp). Out of them, 24,273 contigs (average length of 1087 bp) matched our coverage criteria and were paired with 16,516 *P. glauca* genes (69.7% of the targeted genes) with which they shared at least 95% of identity. On average, 1.47 contigs overlapped each of the 16,516 genes (min 1 - max 17 contigs/gene). Given that the *P. abies* exome capture was conducted with *P. glauca* probes, the success of the approach depended mainly on the degree of sequence identity between species.

### SNP detection and distribution

BWA mapped about 18.5 millions of captured sequences against the paired homologous contigs (Fig. 1). PLATYPUS detected 137,534 variants (comprising multiple nucleotide polymorphisms (MNP), including 115,336 SNPs. The GS REFERENCE MAPPER mapped about 14 millions of captured sequences against the paired homologous contigs and produced 192,449 polymorphisms (comprising MNP) including 191,334 SNPs (Fig. 1). We found 238,666 SNPs that were not in the intersection of the data generated by both PLATYPUS and GS REFERENCE MAPPER but were detected by either of the two softwares, but also 68,004 SNPs predicted simultaneously by the two methods, that represented roughly 60% and 35% of the SNP datasets predicted by PLATYPUS and GS REFERENCE MAPPER, respectively. Out of them, 61,771 SNPs met the in-house quality filters (detailed in Methods) and consisted in the *P. abies* high-confidence SNP resource (Additional file 1). These SNPs were all non-singletons with an average depth of 183 (median = 103) and an average minor allele frequency (MAF) of 0.31 (median = 0.32). The 61,771 SNPs were distributed among 18,587 contigs, representing 13,543 *P. glauca*-homolog SNPed genes [30], for an average of 4.56 SNPs per SNPed gene. Among the 16,516 *P. abies* genes uniquely matched to the GCAT3.3 *P. glauca* gene catalog, 2973 (18%) had no high-confidence SNPs. These genes are technically qualified as unSNPed in the limits of the present study and criteria used to retain only high-confidence SNPs. When these were considered with SNPed genes, an average of 3.74 SNPs per gene was obtained. The SNP abundance was 0.234 SNP per 100 sites or one SNP per 427 sites, when considering the 13,543 SNPed genes only. When the total of 16,516 genes including 2973 unSNPed genes was considered, the corresponding numbers were 0.219 SNP per 100 sites and one SNP per 457 sites.

Because of the high synteny and collinearity among Pinaceae and especially among spruce genomes [9, 33–35, 53], a proxy for the genomic position of 5391 *P. abies* genes was used by determining the position of their *P. glauca* homologs on the recently augmented *P. glauca* genetic map [9] (Additional file 1). These 5391 genes were largely spread on the 12 chromosomes of *P. glauca.* Given that the number of genes is quite homogeneous across the 12 spruce chromosomes [53, 54], the 12 *P. abies* chromosomes appear all well represented in the present SNP catalog.

### Validating the SNP resource with a genotyping array

Out of the 6000 SNPs selected to construct the SNP genotyping array, 5660 (94.3%) were successfully manufactured (Additional file 2), whereas the Illumina probe synthesis failed for the remaining 340 SNPs (5.6%), which is well within previously reported rates of manufacture failure [27, 39, 40]. The 5660 successfully manufactured SNPs included 5571 predicted SNPs from exome sequencing and 89 control SNPs used successfully for genotyping in previous SNP arrays. From the initial number of 5660 SNPs successfully manufactured, 4768 were deemed valid, corresponding to an overall success rate of 84.2% (Table 1), and representing as many distinct genes with annotated homologs in the *P. glauca* catalog of transcribed genes [30]. All 4768 SNPs had a call rate ≥ 80%, and the average call rate was 99.3%. According to the two positive controls included on each genotyping plate, the internal reproducibility of the SNP array was estimated at 99.94%. The success rate for the control SNPs recycled from previous white spruce SNP genotyping arrays reached 85.4%, which was only slightly higher than that of newly predicted Norway spruce SNPs (true positive rate = 84.2%; Table 1). Out of the 892 failed SNPs, 310 SNPs were monomorphic (all individuals clustered in a single homozygous class), 508 SNPs resulted from probes likely annealing to paralogous loci (*F*_*e*_ ≥ 0.80), and 74 SNPs showed no clear clustering in two or three expected genotypic classes or weak signal intensity. The rate of failed SNPs was also comparable between control and predicted SNPs (Table 1).

**Table 1:** Genotyping success rate of the *Picea abies* Infinium SNP array and true positive rate according to sources of SNPs

Source of SNPs	Number of successfully manufactured SNPs	Segregating SNPs		Failed SNPs			
		Number of segregating SNPs	Genotyping success rate/true positive rate^a^	Number of monomorphic SNPs	Number of paralogous SNPs^c^	Number of other non-segregating SNPs^d^	Total number
Control SNPs recycled from previous genotyping arrays	89	76	85.4%	6	4	3	13 (14.6%)
Newly predicted SNPs^b^ from exome capture and sequencing	5571	4692	84.2%	304	504	71	879 (15.8%)
Total	5660	4768	84.2%	310	508	74	892 (15.8%)

^a^Genotyping success rate for control SNPs, and true positive rate for newly discovered SNPs from exome capture and sequencing

^b^SNPs identified among the 61,771 SNPs predicted by both PLATYPUS and GS REFERENCE MAPPER and satisfying the quality filters detailed in Materials and Methods

^c^Paralogous SNPs, those with high excess of heterozygotes with *F*_*e*_ ≥ 0.80

^d^Other non-segregating SNPs, those showing no clear clustering in two or three expected genotypic classes, or showing weak signal intensity

### Distribution of SNP abundance across gene functional categories

**Fig. 2:**
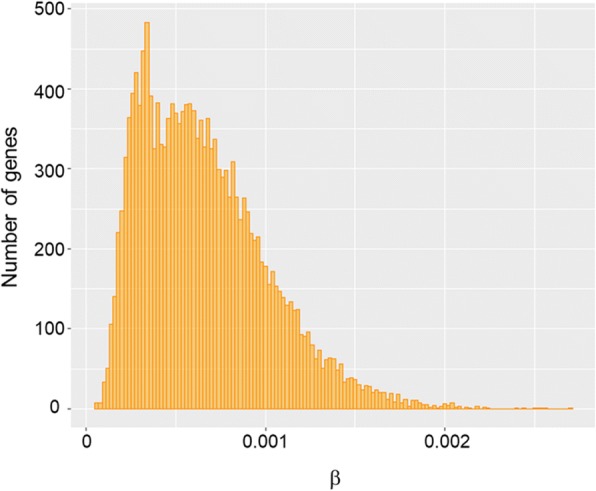
Distribution of SNP abundance across genes. β is the computed SNP abundance parameter correcting for variable sequencing depth among genes, which was used to compare SNP abundance among them

Because observed values of SNP abundance were correlated with sequencing depth, we estimated for each contig the β parameter which corrects for this bias (see Methods). We then used this parameter to compare SNP abundance among the 16,516 genes carrying high-confidence SNPs. The distribution of β values was right-skewed with fewer genes harboring high SNP abundance (Fig. 2). We looked at the annotations of the 30 most SNPed genes, those harboring the highest β values of SNP abundance (Additional file 3). As a general trend, this subset of genes was characterized by high functional diversity. Notably, it encompassed seven plant disease resistance genes involved in stress response to biotic and abiotic stresses: a phytanoyl-CoA dioxygenase gene, two genes encoding cell wall-degrading enzymes, a gene encoding the 26S proteasome, a gene belonging to the Leucine Rich Repeat family, an UDP-glycosyltransferase gene and a heat shock protein class III gene.

Functional annotations (GO terms, gene families, orphans, and conifer-specific genes) of the 10% most SNPed genes (those with highest β values) were then compared with those of the remaining dataset. Across GO terms, 10 Molecular Functions (MF), 15 Biological Processes (BP) and 5 Cellular Components (CC) were significantly enriched in the highly polymorphic gene subset (*P* < 0.05) (Table 2). The most significant (*P* < 0.01) MF were endoribonuclease activity and hydrolase activity; the most significant BP were alcohol metabolic process, response to insect, and ER to Golgi vesicle-mediated transport; and the most significant CC were plant-type vacuole membrane, cell wall, and anchored component of membrane (Table 2). Additional file 4, which illustrates the hierarchical relationships among these significant GO terms, highlighted an interesting pattern within the MF category. Indeed, three general terms were significantly enriched (namely transferase activity, hydrolase activity, and oxidoreductase activity), and all of them were grouped under the umbrella « catalytic activity » (Additional file 4: Figure S1). In addition, hydrolase activity also included three significantly enriched terms: aspartyl esterase activity, pectinesterase activity and endoribonuclease activity.

**Table 2:** GO terms significantly enriched among the 10% genes with highest SNP abundance following Fisher's exact tests

GO ID	Term	*p*-value
Molecular function		
GO: 0004521	Endoribonuclease activity	0.0029
GO: 0016787	Hydrolase activity	0.0096
GO: 0016229	Steroid dehydrogenase activity	0.0106
GO: 00016757	Transferase activity, transferring glycosyl groups	0.0120
GO: 0045330	Aspartyl esterase activity	0.0157
GO: 0015299	Solute: proton antiporter activity	0.0157
GO: 0015491	Cation: cation antiporter activity	0.0174
GO: 0005507	Copper ion binding	0.0229
GO: 0030599	Pectinesterase activity	0.0277
GO: 0016491	Oxidoreductase activity	0.0409
Biological process		
GO: 0006066	Alcohol metabolic process	0.0026
GO: 0009625	Response to insect	0.0053
GO: 0006888	ER to Golgi vesicle-mediated transport	0.0081
GO: 0007049	Cell cycle	0.0190
GO: 0015804	Neutral amino acid transport	0.0217
GO: 0015980	Energy derivation by oxidation of organic compounds	0.0222
GO: 0010351	Lithium ion transport	0.0225
GO: 0006364	rRNA processing	0.0247
GO: 0010015	Root morphogenesis	0.0277
GO: 0009718	Anthocyanin-containing coumpound biosynthetic process	0.0294
GO: 0006820	Anion transport	0.0400
GO: 0009962	Regulation of flavonoid biosynthetic process	0.0409
GO: 0046189	Phenol-containing compound biosynthetic process	0.0411
GO: 0016458	Gene silencing	0.0422
GO: 0043269	Regulation of ion transport	0.0460
Cellular component		
GO: 0009705	Plant-type vacuole membrane	0.00082
GO: 0005618	Cell wall	0.00495
GO: 0031225	Anchored component of membrane	0.00786
GO: 0009504	Cell plate	0.02325
GO: 0010319	stromule	0.04399

**Fig. 3:**
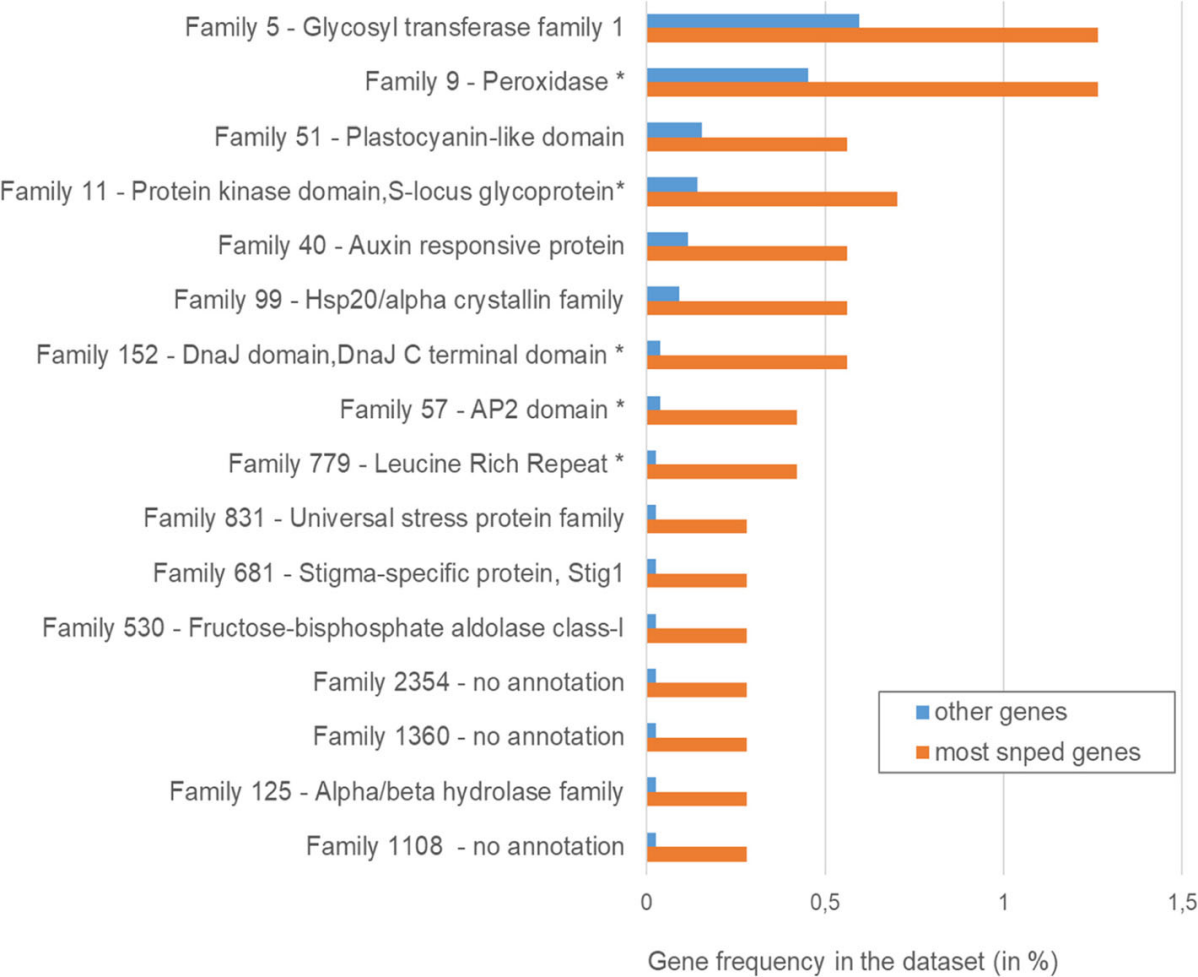
Differential representation of gene families among the 10% most SNPed genes versus the other genes. The 16 families found differentially represented after Fisher’s exact test (*P* < 0.05) are represented; the stars (*) indicate the statistically most significant differences (*P* < 0.01). Gene family identifiers were retrieved from the ConGenIE database (http://www.congenie.org)

After correction for multiple testing, none of the 69 gene families tested appeared differentially distributed between the 10% most SNPed genes and the rest of the dataset. However, based on uncorrected *p*-values, 16 families were significantly more represented within the most SNPed genes (5 families being significant at *P* < 0.01 and 11 others at *P* < 0.05) (Fig. 3). Notably, 7 out of these 16 gene families appeared involved in response to biotic or abiotic stresses based on annotations retrieved from the ConGenIE database.

Conifer-specific genes were significantly more represented in the 10% most SNPed genes (7.5%) than in other genes (5.3%) (Fisher’s exact test, *P* = 0.007), but no significant enrichment was observed for orphans (1.5 and 2.3%, respectively) (Fisher’s exact test, *P* = 0.06).

## Discussion

### De novo exome reference assembly and SNP detection

Using *P. glauca* probes for *P. abies* exome capture, the target recovery rate obtained (74.5%) was comparable to that previously obtained for *P. mariana* (75.9%) using the same set of probes [40]. When discarding contigs with extremely high or low coverage (see Methods), the final recovery rate (69.7%) was slightly lower, but it remains high considering the size and complexity of the *Picea* genome, and that the capture tool was originally designed on a phylogenetically distant species, *P. glauca* [50, 55].

The *P. abies* SNP resource developed herein includes 61,771 high-confidence SNPs distributed over 13,543 genes, which represent almost half of the predicted genes from genome sequencing [2]. While the number of genes represented in this resource is comparable to that obtained in *P. mariana* using a similar approach (14,909 genes) [40], the number of predicted high-confidence SNPs was smaller. This trend was expected because the number of trees used (10) for exome capture/sequencing and ensuing discovery of SNPs was smaller than that used previously in *P. mariana* (44). Hence, it is likely that SNPs with low MAF in natural populations were not well represented in our sampling. However, these SNPs are less informative for most common genomic applications (e.g. genomic selection, landscape genomics, association mapping) [54].

### Validating the SNP resource with a genotyping array

The true positive rate obtained for *P. abies* predicted SNPs (84.2%) was lower than that of *P. mariana* (96.3%) [40] and *P. glauca* (92.1%) [26], but higher than that reported for *Pseudotsuga menziesii* (72.5%) [39], using the same genotyping platform. However, the true positive rate of predicted SNPs (84.2%) was only marginally lower than the genotyping success of *P. abies* control SNPs recycled from previous genotyping arrays (85.4%), suggesting that the SNP discovery pipeline was reliable, and that much of the failure rate could be attributable to the genotyping assay. Indeed, the genotyping success rate of control SNPs reported herein was marginally lower than that obtained with previous Infinium genotyping arrays for various spruce species (e.g. 96.7% for *P. mariana* [40]; between 90.7% and 95.4% for *P. glauca* [28]). The main difference between the present and previous studies is that the validation of predicted *P. abies* SNPs relied on a large pre-manufactured maize Infinium iSelect array, rather than a custom-made Infinium iSelect array for spruce-only SNPs. Thus, it is possible that a number of non-specific maize probes hybridized partially to spruce loci (and vice versa) and increased the failure rate of both predicted and control spruce SNPs. This observation is supported by the fact that a majority of failed control SNPs (7 out of 13, see Table 1) showed segregating patterns indicative of differential probe hybridization efficiency and specificity (i.e. probes annealing to paralogous sequences, or to sequences carrying additional polymorphisms [56]). The remaining failed control SNPs were monomorphic, which could indicate true monomorphism in the breeding population used for SNP validation.

### SNP abundance across enriched functional categories

A variety of GO terms and gene families significantly over-represented among the 10% most SNPed genes appeared to be related to stress response, and thus, of particular interest for future population genomic investigations. Within molecular functions, hydrolases, oxidoreductases and transferases were the most represented enzymes, in line with the results of a large-scale climate adaptation study in white spruce [23]. In addition to the general GO term hydrolase activity, hydrolases comprised three specific GO terms that were significantly enriched among the 10% most SNPed genes: endoribonuclease, aspartyl esterase, and pectinesterase, which all include genes generally related to stress response. For instance, the GO term endoribonuclease activity was shown to be related to defense response against a variety of pests in rice [57], while pectinesterase was reportedly involved in defense responses against pathogens [58] and leaf senescence in rice [59]. The aspartyl esterase GO term includes a dicer-like protein encoded gene associated with epigenetic regulation and RNA-mediated gene silencing in plants under environmental stresses [60]. High-throughput sequencing of small RNA sequences in *Pinus contorta* also revealed the presence of a novel dicer-like family specific to conifers, and responsible for changes in small RNA expression [61]. In Norway spruce specifically, the dicer-like genes *PaDCL1* and *PaDCL2* were found differentially expressed in families produced under contrasted embryogenesis temperature / photoperiod conditions, suggesting the involvement of these genes in epigenetic regulation of spruce development [62].

Other lines of evidence supported hydrolase-encoding genes as potent candidates for population genomic investigations in relation to adaptation. For instance, the alpha/beta hydrolase family was found over-represented in the 10% most SNPed genes (Fig. 3), while this gene family was reported to be involved in adaptation to salinity stress in *Thellungiella*, a plant closely related to *Arabidopsis* [63]. In addition, three hydrolase-encoding genes were found among the ten most polymorphic genes (Additional file 3). Among them, two genes encoding cell wall-degrading enzymes belonged to the glycosyl hydrolase family [64], a gene family previously reported as highly polymorphic in *P. glauca* [26] and *P. abies* [65]. A gene encoding a hydrolase protein was also reported as a top candidate for local adaptation in a study investigating convergent adaptation in lodgepole pine (*Pinus contorta*) and interior spruce (*P. glauca* × *P. engelmannii*) [24].

Along with hydrolases, transferases were the most represented enzymes, in agreement with the results of a climate association study conducted in *P. glauca* [23]. For instance, the GO term transferase activity - transferring glycosyl groups was over-represented among the 10% most SNPed genes (Fig. 3), as well as genes belonging to the large family glycosyl transferase gene family 1. Glycosyl transferases are thought to play important roles in plant defense responses to stress by glycosylating secondary metabolites [66]. Accordingly, the GO term transferase activity - transferring glycosyl groups was previously shown to include genes associated with drought stress in wheat [67], while the glycosyl transferase gene family 1 was previously reported as over-represented in genes under diversifying selection in *P. abies* and *P. glauca* [65], and is involved in response to *Fusarium* infection in wild potato [68] and wheat [69]. Similarly, a gene belonging to the same glycosyl transferase family 1 cited above was found among the 30 most polymorphic genes in the present study (Additional file 3). In addition to glycosyl transferases, genes belonging to methyltransferases also appeared highly polymorphic. For instance, the second most SNPed gene across our dataset (Additional file 3) was a transferase encoding an S-adenosylmethionine-dependent methyltransferase, which was reportedly involved in oxidative stress in the ascomycete *Podospora anserina* [70]. The involvement of methyltransferase genes in local adaptation pathways has also been reported in lodgepole pine and interior spruce [24].

In addition to the general GO term oxidoreductase activity, the GO term steroid dehydrogenase activity, which groups under the umbrella “oxidoreductase activity”, was found enriched in the 10% most SNPed genes (Additional file 4). While oxidoreductase activity includes an array of genes involved in stress response, steroid dehydrogenase activity was associated to genes involved in environmental interactions such as defense against biotic agents and adaptation to abiotic stresses [71]. We also found a glutamate dehydrogenase gene among the 30 most polymorphic genes (Additional file 3), which was shown to be up-regulated in *Arabidopsis thaliana* under stress conditions [72].

Among the 10% most SNPed genes, the most significant enriched biological processes was alcohol metabolic process, which includes genes shown to be involved in salt stress in chickpea [73]. The second most significant biological process was response to insect, which is directly related to biotic stress response.

Several other gene families and genes involved in stress response showed high level of polymorphism in our dataset, although they were not associated significantly with specific GO terms. For instance, the leucine rich repeat (LRR) and protein kinases gene families were over-represented in the 10% most SNPed genes (Fig. 3), in line with results from previous studies in spruces [23, 24, 65]. A LRR gene was also found among the 30 most polymorphic genes (Additional file 3), while high SNP abundance in NBS-LRR genes has been previously observed across different plant genomes [74–79]. In addition, genes coding for heat shock proteins (HSP) were well represented among the 10% most SNPed genes. These genes likely represent good candidates for population genomics studies in relation to adaptation, as they play a crucial role in protecting plants against abiotic stresses [80]. Their involvement in local adaptation pathways was suggested for lodgepole pine and interior spruce [24]. Two gene families, the HSP40/DnaJ chaperones and the Hsp20/alpha crystalline family, were over-represented in the 10% most SNPed genes (Fig. 3), and a HSP class III gene belonging to the Hsp20/alpha crystalline family was also found among the 30 most SNPed genes (Additional file 3). Both HSP families were reported as highly polymorphic in *Picea sitchensis* [81], and the Hsp20/alpha crystalline family was also found highly polymorphic in *P. glauca* [26]. Finally, the most SNPed gene across the whole dataset was found to encode a Phytanoyl-CoA dioxygenase (Additional file 3), which was previously shown to be involved in the electron transfer in *Eucalyptus* resistance response against *Cylindrocladium* [82].

Plant response to biotic and abiotic stresses is expected to by tightly linked to the adaptive potential of individuals in natural populations [83]. This is especially true for long-lived plants with long generation times, such as spruces, that have to cope with a range of changing environmental conditions throughout their life cycle before even reaching sexual maturity. In such situation, high levels of genetic polymorphism may likely indicate the action of diversifying selection. Hence, the observed distribution of genetic polymorphisms across functional gene categories should be useful to identify candidate genes and gene families for future population genomic studies in relation to adaptation.

## Conclusion

This work demonstrates the efficiency of exome capture combined with Illumina MiSeq sequencing to generate a robust gene sequence assembly and a catalog of annotated high-confidence gene SNPs in a species with a challenging large genome. This catalog  represents a valuable genomic resource, being representative of over 13 K genes distributed across the *P. abies* genome. It will be helpful to validate data obtained from GbS and should serve a variety of population genomic studies and breeding applications in Norway spruce. Because of SNP and gene annotations, it should also facilitate comparative genome mapping, association mapping and landscape genomic studies with other spruce and conifer species.

## Methods

### Plant material and DNA extractions for exome capture

Fresh needles were collected from 10 Norway spruce (*Picea abies* [L.] Karst.) grafted trees sampled in a 27-year old breeding orchard located north of Quebec City (Natural Resources Canada). All trees originated from central Europe, six of them being representative of distinct natural populations from Poland (3), Belorussia (1), and Latvia (2), and the four remaining ones being of unknown location. No permit was required to collect tissue in any location sampled in this study. DNA was isolated from needles using the Qiagen DNeasy Plant Mini Kit (Mississauga, ON, Canada) and quantified using the PicoGreen fluorescent dye (Invitrogen). Afterward, DNA samples were assembled in two pools of five individuals with equimolar concentrations [84]. In order to generate a reference sequence assembly with minimum genetic polymorphism, DNA was also extracted from a haploid megagametophyte, followed by whole-genome amplification using the WGA2 kit (Sigma-Aldrich, Oakville, ON, Canada).

### Probe design for exome capture, target enrichment and sequencing

Probes were designed from *P. glauca* transcriptome sequences [30] and were already used successfully under an exome capture framework on *P. glauca* [85] and *P. mariana* [40]. About 20 probes ranging from 50 to 105 nucleotides were designed for each transcript with each base being covered by two probes on average [40]. To capture their *P. abies* homologs on the two DNA pools and the haploid megagametophyte described above (Fig. 1), we used a liquid-phase capture (SeqCap EZ developer, IRN 6089042357, OID35086, Roche Nimblegen) that targeted 23,684 genes (0.5 M probes), followed by an Illumina MiSeq paired-end sequencing. MiSeq was used because it generates relatively long reads (300 bp). For each pool and the megagametophyte, one microgram of DNA was used to prepare TruSeq gDNA libraries (Illumina, San Diego, CA) according to the manufacturer’s instructions. Libraries (600-bp mean insert size) were amplified by ligation-mediated PCR using platform specific primers, as described in the NimbleGen SeqCap EZ Library LR User’s guide (Roche NimbleGen, Madison, Wisconsin). Emulsion PCR and MiSeq sequencing were performed according to manufacturer’s instructions at the sequencing platform of the Institute for Integrative Systems Biology (Univ. Laval, Québec, Canada).

### De novo exome reference assembly and pairwise sequence comparisons

All paired megagametophyte reads were submitted to a de novo assembly by using the A5 assembler software [86] with default parameters (minimum read length = 35 bp and k-mer size = 35 bp) (Fig. 1). The resulting contigs were blasted against the *P. glauca* coding sequences [30] which were originally used to design the probe sequences. Only *P. abies* contigs matching *P. glauca* transcripts with a minimum threshold (95% of sequence identity and blastn e-value <1e^− 5^) and an average coverage between 25 and 800 were retained for subsequent steps.

### Reference-guided alignment and SNP detection

Two protocols were used for the alignment of pool reads and the SNP detection, producing two SNP datasets over the reference-guided alignment (Fig. 1). In the first protocol, reads were aligned with BWA (Burrows-Wheeler Alignment) using a minimum seed length of 33 bp, a mismatch penalty of 10 and a gap open penalty of 100. SNPs were detected with PLATYPUS using the following criteria: minReads = 25, maxVariants = 2, minMapQual = 10, minBaseQual = 10, minGoodQualBases = 10, badReadsThreshold = 10, rmsmqThreshold = 20 and hapScoreThreshold = 15. The second protocol used the GS REFERENCE MAPPER software (version 2.8; 454 Life Science) for both alignment and SNP detection with the following parameters: minimum read length = 40 bp, seed step = 12 bp, seed length = 16 bp, seed count = 3 bp and 99% of minimum overlap identity.

Lastly, two Python scripts (https://www.python.org/) were developed to identify and retain SNPs common to both datasets, and to extract the 100 bases upstream and downstream of each SNP (or shorter when the SNP was too close to a contig end). Only SNPs satisfying the following in-house criteria were included in the SNP resource: MQ (root-mean-square mapping quality) ≥ 20, MMLQ (median minimum base quality for bases around variant) ≥ 10, QD (quality by depth) ≥ 10, PP (posterior probability) ≥ 20, SbPval (binomial *P-value* for strand bias test) ≥ 0.01, hap score ≥ 15, max GOF (max allowed value for goodness-of-fit test) ≥ 20, SC (sequence context) ≥ 0.95, and a minimum of two reads for the alternative allele. Thus, singleton SNPs were de facto excluded from the resource in order to minimize the rate of false positives. Furthermore, only bi-allelic SNPs were retained since they are abundant and easier to genotype with common high-throughput genotyping platforms, and given that multi-allelic SNPs are more likely to result from variation at paralogous loci [40, 87].

### Genotyping assay

An Infinium iSelect SNP array (Illumina, San Diego, CA) was developed to estimate the true positive rate for a subset of newly identified SNPs, and to genotype trees for future population genomic applications. The array consisted of 6000 beads, with use of type II SNPs (one bead per SNP) [88] and one SNP per gene to maximize the number of SNPs and gene loci on the chip. Two subsets of SNPs were submitted for manufacturing: 5907 newly predicted SNPs and 93 control SNPs previously genotyped successfully with two GoldenGate (Illumina) SNP arrays [5, 17].

Newly predicted SNPs that were included in the array had to satisfy the following criteria: i) no SNP or indel within the 50 bp upstream or downstream of the predicted SNP (to ensure a good match of the Infinium probe); ii) a minimum distance of 20 bp from both contig ends (for possible reuse with other genotyping technologies; iii) and an Illumina functionality score ≥ 0.60.

To assess the true positive rate of newly predicted SNPs, a set of 1130 full-sib progenies resulting from various crosses among 35 parents of the *P. abies* breeding population from the Ministère des Forêts, de la Faune et des Parcs of Québec were genotyped with the SNP array. Two samples were used as positive controls and replicated on each 96-well plate to evaluate intra-assay genotyping reproducibility. DNA was isolated from needles and terminal buds by using the DNeasy 96 Plant Kit of Qiagen (Mississauga, Ontario) and following the manufacturer’s instructions.

The SNP genotyping assay was manufactured and carried out at the Génome Québec Innovation Centre (team of Daniel Vincent and François Bacot at McGill University, Montréal, Canada) according to Illumina’s protocols. A minimum of 80 ng of template gDNA per sample was used. Genotype calling was conducted using the GENOME STUDIO 2.0 software (Illumina). All SNPs with a GenTrain score ≥ 0.13 and a call rate ≥ 80% (average call rate = 99.3%) were visually inspected in GENOME STUDIO, and manually cured to reject monomorphic and non-segregating polymorphisms. In addition, polymorphisms with large excess of heterozygotes (*F*_*e*_ ≥ 0.80) were discarded as they usually result from probes annealing to paralogous loci.

### SNP abundance

SNP abundance was estimated as the number of SNPs observed within a contig, divided by contig length. Because this diversity parameter was correlated with contig depth (Pearson’s correlation *r* = 0.30), we estimated the beta (β) parameter developed by Novaes et al. [89], which corrects for sequencing depth using the following formula:

1 $$ \beta =\left[\left(S+1\right)/L\right]/\left[{\sum}_{i=1}^{D-1}\ \left(1/i\right)\right] $$

where *S* is the number of SNPs detected in the contig, *L* is the contig sequence length and *D* is the average depth for the contig (i.e. the average number of reads covering a nucleotide position). Given that the correlation between β and contig depth was low with *r* = 0.13, this parameter was deemed appropriate to compare SNP abundance across genes. When a gene was composed of multiple contigs, a weighted average β based on sequence length was computed for the gene (Additional file 5).

### Gene annotation

The *P. abies* contigs were paired with the *P. glauca* coding sequences [30] from which the probes for exome capture were designed, and with the sequences of predicted genes based on the *P. abies* whole-genome sequence [2]. Gene annotations, namely GO accessions, gene families, orphans (i.e. gene not included in any family), and conifer-specific genes, were then inferred from homologous gene sequences (minimum sequence identity level of 98%) using the ConGenIE public database (available at http://www.congenie.org) (Additional file 5).

We performed enrichment tests between the 10% most SNPed genes (those with the highest β values) and the remaining genes for the following functional categories: GO terms, gene families, orphans (i.e. genes with unknown gene families), and conifer-specific genes.

We used the package topGO [90], available in R BIOCONDUCTOR [91], to assign genes to GO terms, and to test whether the 10% most SNPed genes were significantly enriched in some GO terms. The initial gene set consisted of the 5735 genes, among which 4342 genes were associated with a molecular function, 4302 genes were associated with a biological process, and 3572 genes were associated with a cellular component. Methods implemented in topGO compute the significance of a GO term enrichment based on its neighborhood [90]. We applied the *weight01* method, which is a mixture of the *elim* and the *weight* methods, both taking into account the GO hierarchy [90]. GO terms with less than five genes were excluded (nodesize = 5), and Fisher’s exact tests were applied to assess statistical significance. Non-adjusted *p*-values were used, as commonly done in similar studies (e.g. [92]) and as recommended in the topGO user guide (available at http://bioconductor.org/packages/3.7/bioc/vignettes/topGO/inst/doc/topGO.pdf).

We then assessed whether some gene families were over-represented within the 10% most SNPed genes, relative to the remaining dataset. The 69 gene families represented by at least two genes in the 10% most SNPed genes were tested for enrichment using Fisher’s exact tests. These tests were also used to determine if orphan genes and conifer-specific genes were over-represented within the 10% most SNPed genes.

## Additional files

Additional file 1: Description of the *Picea abies* predicted SNP resource including quality parameters. (XLSX 13017 kb)

Additional file 2: Description of *Picea abies* SNPs successfully genotyped with the Infinium SNP array. (XLSX 1081 kb)

Additional file 3: The 30 most SNPed genes among the 16,516 *Picea abies* genes analysed. (XLSX 15 kb)

Additional file 4: The subgraph representing the most significant GO terms found by the weighted model produced by TopGO for scoring GO terms for enrichment. Boxes indicate significant terms and box color represents relative significance, ranging from dark red (most significant) to light yellow (least significant). Each shape provides GO term accession, definition, the raw *p*-value and observed frequency. (ZIP 136 kb)

Additional file 5: Annotation of the 16,516 *Picea abies* genes according to GCAT and ConGenIE database with GO accessions. (XLSX 1731 kb)

## Abbreviations

BP: Biological Process
BWA: Burrows-Wheeler Alignment
CC: Cellular Component
ENA: European Nucleotide Archive
EST: Expressed sequence tag
EVA: European Variation Archive
*F*_*e*_: Excess of heterozygotes
GbS: Genotyping-by-sequencing
GO: Gene Ontology
GOF: Goodness-of-fit
GS: Genomic selection
GWAS: Genome-wide association studies
HSP: Heat shock proteins
LRR: Leucine rich repeat
MAF: Minor allele frequency
MF: Molecular Functions
MMLQ: Median minimum base quality for bases around variant
MNP: Multiple nucleotide polymorphisms
MQ: Root-mean-square mapping quality
PP: Posterior probability
QD: Quality by depth
QTL: Quantitative trait loci
RNA-seq: RNA sequencing
SbPval: Binomial *P-value* for strand bias test
SC: Sequence context
SNP: Single nucleotide polymorphism
vcf: Variant call format
WGA: Whole-genome amplification
