# Supplementary figures and images for: A catalog of annotated high-confidence SNPs from exome capture and sequencing reveals highly polymorphic genes in Norway spruce (Picea abies)
Source: BMC Genomics. 2018 Dec 17;19:942. doi: 10.1186/s12864-018-5247-z (PMC6296092; doi:10.1186/s12864-018-5247-z)

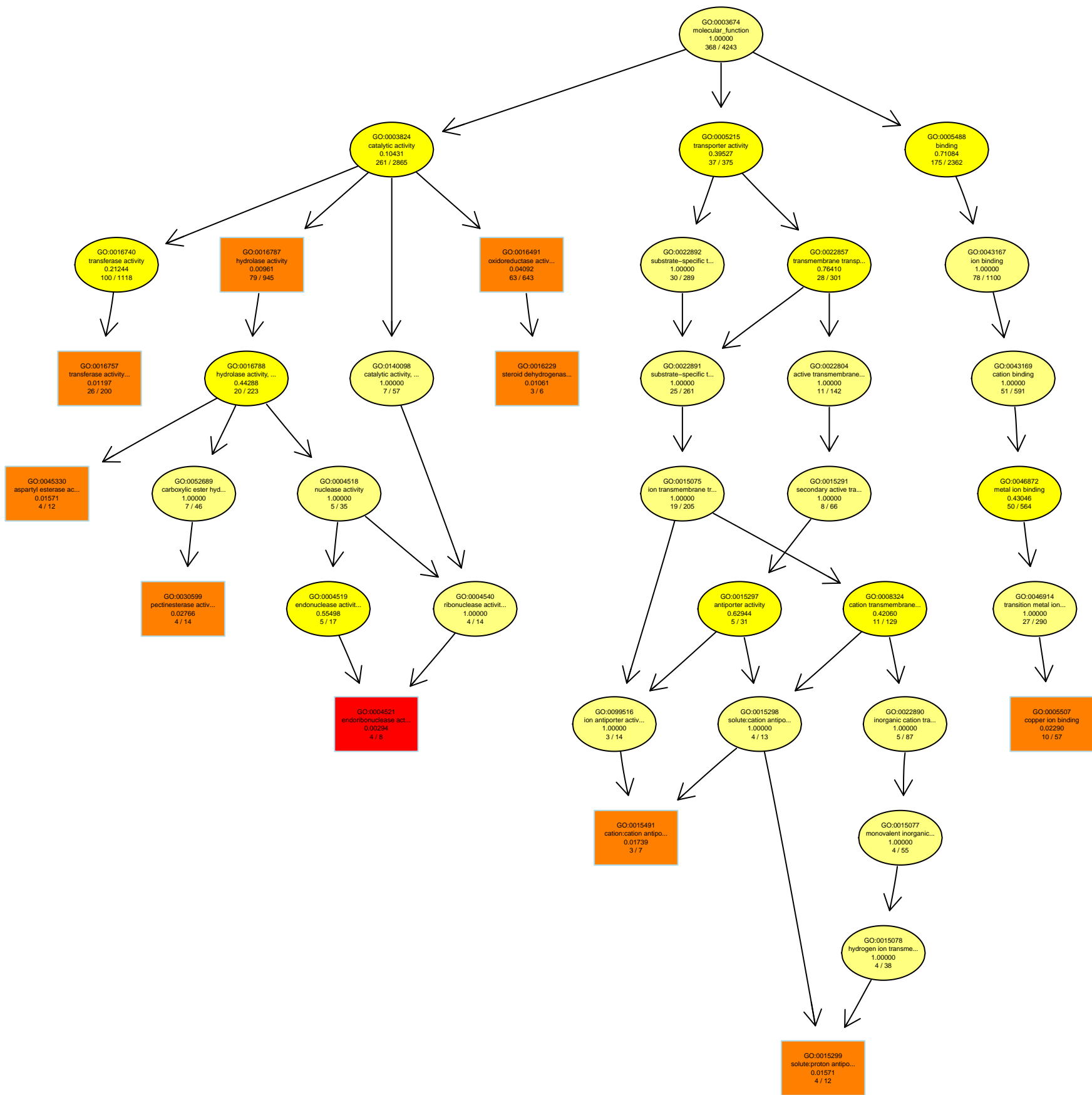

Supplement: Supplementary file 4 — The subgraph representing the most significant GO terms found by the weighted model produced by TopGO for scoring GO terms for enrichment. Boxes indicate significant terms and box color represents relative significance, ranging from dark red (most significant) to light yellow (least significant). Each shape provides GO term accession, definition, the raw p-value and observed frequency. (ZIP 136 kb) [file 12864_2018_5247_MOESM4_ESM.zip › FigureS1-MF.pdf]

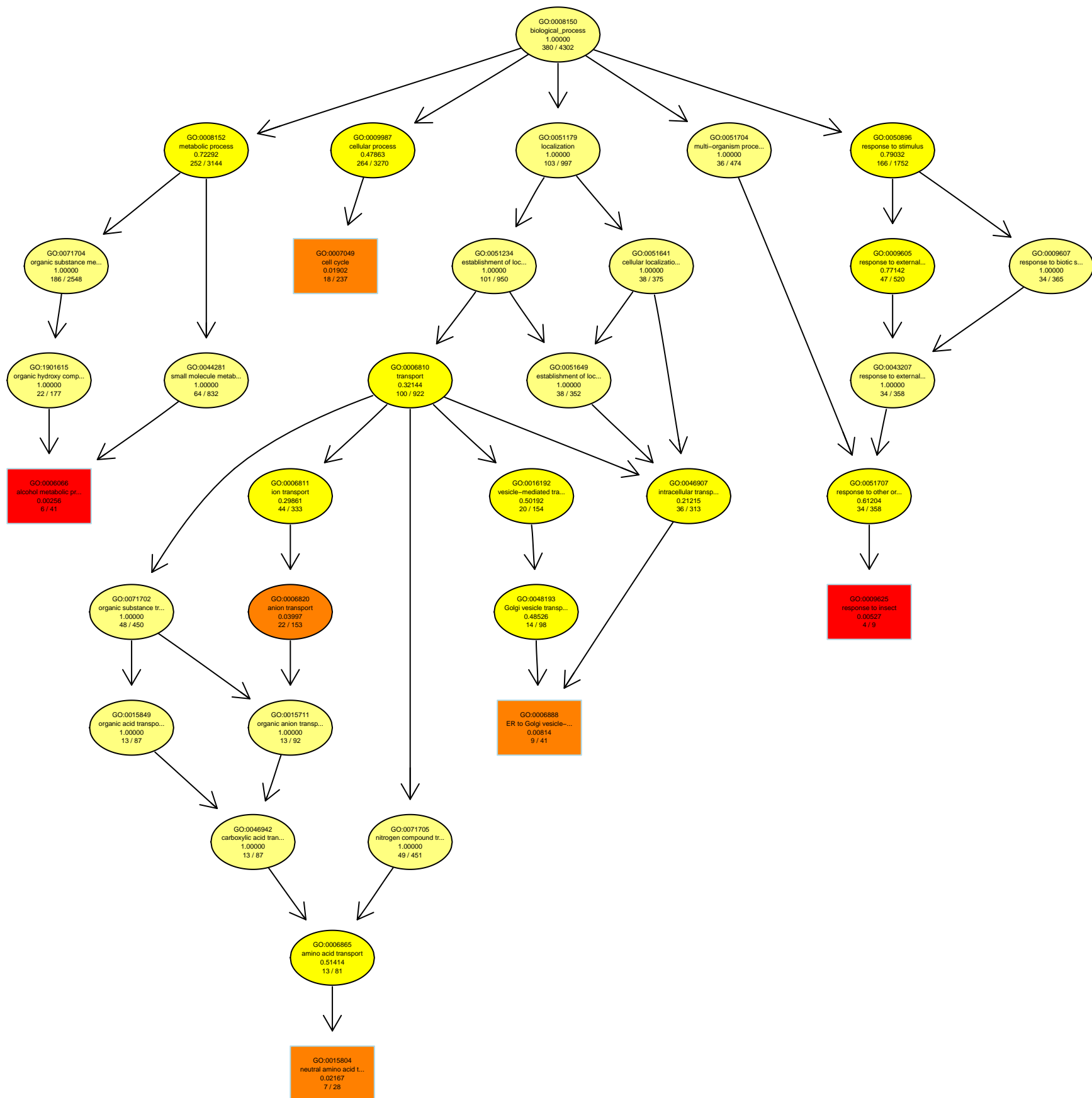

Supplement: Supplementary file 4 — The subgraph representing the most significant GO terms found by the weighted model produced by TopGO for scoring GO terms for enrichment. Boxes indicate significant terms and box color represents relative significance, ranging from dark red (most significant) to light yellow (least significant). Each shape provides GO term accession, definition, the raw p-value and observed frequency. (ZIP 136 kb) [file 12864_2018_5247_MOESM4_ESM.zip › FigureS2-BP.pdf]

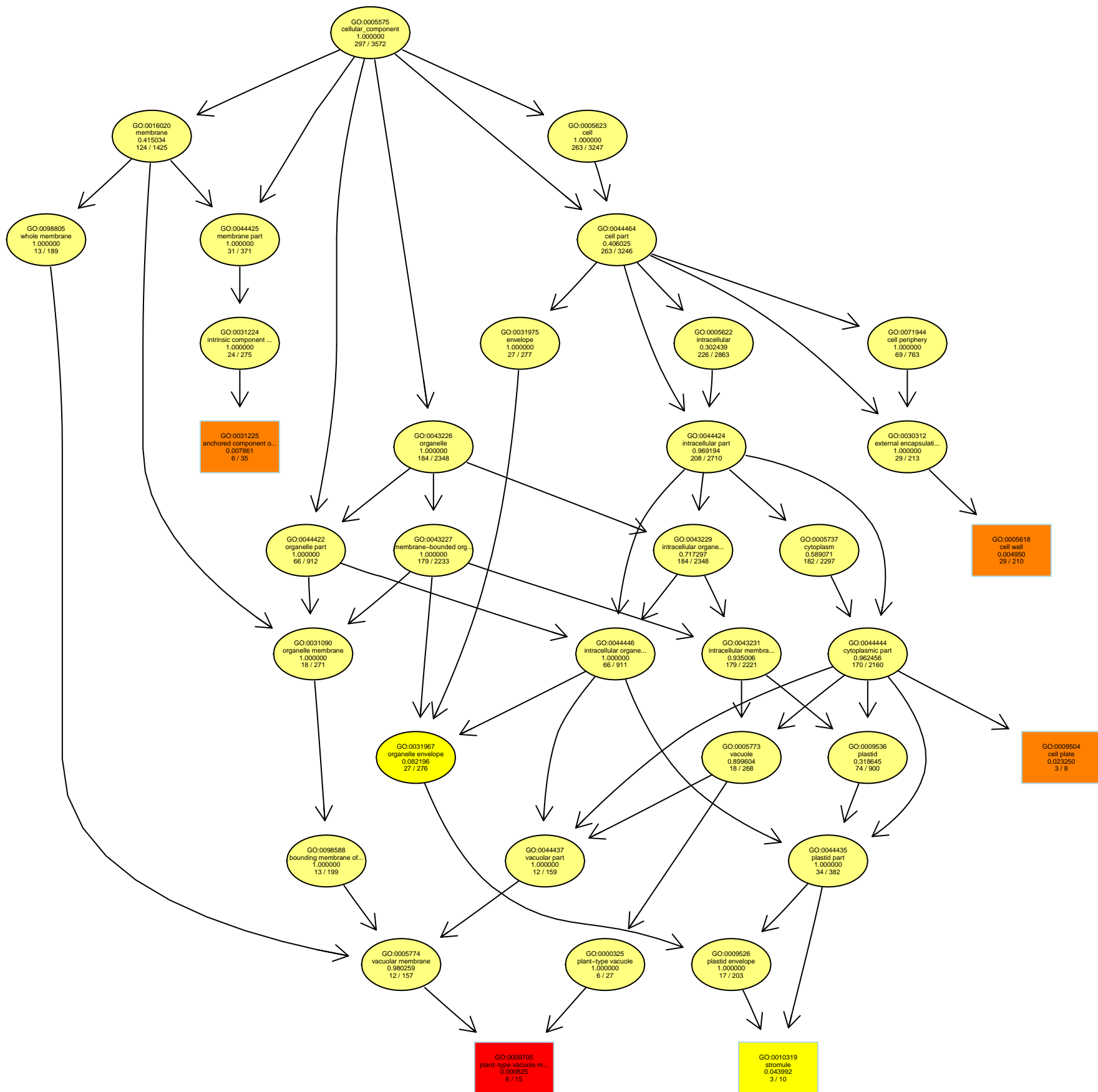

Supplement: Supplementary file 4 — The subgraph representing the most significant GO terms found by the weighted model produced by TopGO for scoring GO terms for enrichment. Boxes indicate significant terms and box color represents relative significance, ranging from dark red (most significant) to light yellow (least significant). Each shape provides GO term accession, definition, the raw p-value and observed frequency. (ZIP 136 kb) [file 12864_2018_5247_MOESM4_ESM.zip › FigureS3-CC.pdf]
